# Endocan may predict the presence of coronary slow flow and coronary artery disease

**DOI:** 10.1590/1806-9282.20240515

**Published:** 2024-09-02

**Authors:** Muhammet Mucip Efe, Aydın Akyüz, Cihan Aydın, Aykut Demirkıran, Şeref Alpsoy

**Affiliations:** 1 Namık Kemal University, Faculty of Medicine, Department of Cardiology – Tekirdağ, Turkey.

**Keywords:** Coronary angiography, Coronary artery disease, Coronary vessels

## Abstract

**OBJECTIVE:**

Coronary artery disease (CAD) is frequent, but coronary slow flow (CSF) is a less common cardiovascular disease with a significant risk of mortality and morbidity. Endocan is a proinflammatory glycopeptide that has been investigated in cardiovascular diseases as well as some inflammatory diseases in recent years. We planned to compare the levels of endocan in both CAD and CSF in a similar population and examine the relationship of endocan with additional clinical variables.

**MATERIALS AND METHODS:**

In the trial, we included 169 consecutive subjects having a coronary angiography indication. According to the results of coronary angiography, 58 people were included in the CAD group, 52 were in the CSF group, and 59 people were in the control group. The control group includes those who did not have any lesions in their epicardial coronary arteries. Thrombolysis in myocardial infarction (TIMI)-frame counts (TFC) were calculated for all patients.

**RESULTS:**

Notably, 2.6% of the population in our study had CSF. Both the CAD (555±223 pg/mL) and CSF (559±234 pg/mL) groups had higher endocan levels than the control group (331±252 pg/mL) (p<0.001). There were similar endocan levels between the CAD and CSF groups. Endocan levels were shown to be favorably associated with mean TFC (r=0.267; p0.001). Serum endocan levels (particularly those above 450 pg/mL) and the presence of hyperlipidemia were the most important predictors of both CAD and CSF.

**CONCLUSION:**

Endocan levels are higher in CAD and CSF patients than in those with normal coronary arteries.

## INTRODUCTION

Coronary slow flow (CSF) is characterized by slower-than-normal blood flow into the distal coronary arteries. It is a well-known but poorly understood form of coronary artery disease; therefore, it is sometimes referred to as the CSF phenomenon^1-3^. CSF, which can be detected during coronary angiography (CAG), is defined as delayed filling of the distal portions of the coronary arteries without significant stenosis of the epicardial arteries. Endocan, which is an endothelial cell-specific protein, is a proteoglycan released from endothelial cells and can be measured in serum. Endocan acts as an adhesion molecule in inflammation-related processes. Therefore, it is considered a key molecule in endothelial dysfunction^4-7^. Endocan may be a biomarker for both lifestyle changes for therapeutic purposes and for follow-up to assess the success of treatments. Some studies show an elevation in endocan levels in CAD and CSF^8-10^. However, there is no study in the literature that compares endocan levels in CAD and CHF in the same cross-sectional study setting.

Considering atherosclerosis and endothelial dysfunction, we hypothesized that endocan may be a potential biomarker associated with CAD and CSF. Therefore, we sought the relationship between serum endocan levels and CAD as well as CSF.

## METHODS

We prospectively enrolled patients (n=1,997) who presented to our institution for a routine follow-up visit to our outpatient clinic with tests suggestive of ischemia and stable angina pectoris and underwent coronary angiography consecutively.

The exclusion criteria were as follows: those with age below 30 years and above 75 years, acute coronary event, moderate or severe valvular dysfunction, acute coronary syndrome, rhythm other than normal sinus rhythm (except for infrequent atrial and ventricular premature beats), and left or right bundle branch block. The exclusion criteria were stent implantation for known CAD and history of coronary bypass surgery, systolic heart failure (left ventricular ejection fraction<50%), chronic or active infection, chronic kidney (glomerular filtration rate [GFR] <60 mL/min) and liver or thyroid dysfunction, and history of malignancy. The body mass index (BMI) was calculated as follows: body weight (kg)/square of height (m²).

### Coronary angiography

Coronary angiography was performed on a Siemens (Artiz Zee, Munich, Germany) device as shown below: coronary arteries were visualized with a 6F or 7F Judkins catheter. Images of the LAD were obtained in at least two positions, left oblique and cranial. The acquisition rate for the planned images was set to 25 image frames/second and the minimum number of images was 80. For each suspected lesion, images were taken in different projections for verification. For this purpose, 6–8 mm of opaque material was manually administered. A total of 50–100 mL of nonionic radio-opaque material was used for each patient.

At least two cardiologists evaluated the coronary anatomy and flow velocity on the archival images. Coronary blood flow velocity and the number of images per second were quantitatively determined as described by Gibson et al., and the reliability of measurements made with this technique has been determined from thrombolysis in myocardial infarction (TIMI) studies^11^. The TIMI frame count values of the arrival of the opaque substance from the ostium to the distal mustache for the LAD artery, from the ostium to the site of separation of the distal posterior branch or distal obtuse margin branch for the circumflex, and from the ostium to the site of separation of the posterolateral branch for the RCA were obtained by finding the picture count values. Normal TIMI frame count (TFC) intervals were determined as 36.28±2.6, 22.28±4.1, and 20.48±3.0 for the LAD, circumflex, and RCA, respectively. These values were found as follows in these studies: normal flow velocities in normal epicardial arteries greater than two standard deviations (presence of CSF means in case of higher TFC than the mean TFC+ 2 x standard deviation) were considered to indicate the presence of CSF. The LAD artery is 1.7 times longer than the circumflex and RCA; therefore, a correction is made for the LAD value. This correction is divided by 1.7 to obtain the TFC of the LAD artery. Mean TFC is the arithmetic mean value of the values obtained from all three arteries. Patients were included in the CAD group if any of the epicardial coronary arteries had a 50% or more reduction in lumen diameter or a 70% or more reduction in area on coronary angiography.

### Blood samples and analysis of endocan levels

Fasting venous blood samples were obtained immediately after the procedure from the patients whose consent was obtained after angiography and who were included in the study.

The samples were immediately centrifuged at 3,000 *g* and stored at -80°C until analysis. Serum endocan levels were measured by enzyme-linked immunosorbent assay (ELISA). Elabscience Human ESM1-Endothelial Cell Specific Molecule 1 (Endocan) ELISA Kit (Cat# E-EL-H1557, Elabscience, Texas, USA) was used for the test. The detection range of the kit was 15.63–1,000 pg/mL. The coefficient of variation in reproducibility was less than 10%. ELISA assays were performed after the reactive agent was prepared by dilution technique.

This cross-sectional study was initiated after obtaining permission from a local ethics committee (30.04.2020 and 2020.77.04.01).

### Statistical analysis

All statistical analyses of the data were performed with IBM^®^ SPSS^®^ Statistics for Mac, Version 20 software (IBM Corp., Armonk, NY).

Continuous variables were presented as either mean±standard deviation or median (min–max). Categorical variables were reported as percentages. Variables were tested for normality of distribution using the Kolmogorov-Smirnov test. The three patient groups were compared by ANOVA for normally distributed variables and the Kruskal-Wallis test for abnormally distributed variables. In case of significant deviations in ANOVA, post hoc analysis was performed using the Tukey test, depending on the homogeneity of variances. Similarly, following the Kruskal-Wallis test, the Dunn test was used in the nonparametric pairwise multiple comparison procedure. Differences between categorical variables obtained as a result of the study were revealed through the "chi-square" test. Spearman or Pearson correlation coefficients were calculated to assess continuous and noncontinuous relationships between biomarkers and other variables. Univariate and multivariate logistic regression analyses were used to determine significant factors affecting CSF. ROC analysis was used for the predictive value of the dependent variable endocan. The statistical significance level for the study was set as p<0.05.

## RESULTS

Patients were similar in terms of demographic characteristics except hyperlipidemia. The CSF and CAD groups had higher rates of hyperlipidemia and higher rates of beta-blocker, aspirin, and statin drug use than the NCA group (p<0.05). LDL-cholesterol and endocan levels were similar in the CSF and CAD groups, but the values of LDL-cholesterol and endocan levels in these two groups were higher than those in the NCA group (p<0.05). The mean number of TFC of the LAD artery, circumflex artery, and RCA was higher in the CSF group compared to the other two groups (p<0.05) (Table 1).

**Table 1:** Demographic, clinical characteristics and laboratory values.

Variables	CSF (n=52)	CAD (n=58)	Control (n=59)	p-value
Age, years	52.9 ± 9.1	53.3 ± 8.5	51.1 ± 8.9	0.156
Gender, male n (%)	39 (75%)	42 (%72.4)	43 (72.9%)	0.949
BMI, kg/m^2^	30.3 ± 4.0	29.3 ± 4.3	28.9 ± 3.8	0.192
Smoking, n (%)	24 (46%)	18 (31%)	28 (47%)	0.139
Diabetes, n (%)	15 (28%)	20 (34%)	12 (20%)	0.228
Hypertension, n (%)	27 (51%)	37 (63%)	32 (54%)	0.403
Hyperlipidemia, n (%)	25 (48%)	20 (34%)	5 (8%)	<0.001
Beta blocker, n (%)	30 (57)	23 (39)	20 (33)	0.033
ASA, n (%)	44 (84)	38 (65)	24 (40)	<0.001
Statin, n (%)	28 (53)	24 (41)	5 (8)	<0.001
CCB, n (%)	11 (21)	11 (19)	8 (13)	0.546
ACEI/ARB, n (%)	22 (42)	31 (53)	27 (45)	0.483
Glucose, mg/dl	122 ± 42	127 ± 54	108 ± 26	0.062
Total cholesterol, mg/dl	195 ± 50	189 ± 48	178 ± 36	0.066
Triglycerides, mg/dl	188 ± 171	186 ± 109	185 ± 138	0.855
HDL cholesterol	43 ± 9	41 ± 11	44 ± 12	0.458
LDL cholesterol	117 ± 36	110 ± 40	96 ± 32	0.016
Uric acid, mg/dl	5.7 ± 1.1	5.1 ± 1.3	5.3 ± 1.3	0063
GFR, ml/dk	92.2 ± 14	94 ± 14	98.6 ± 13	0.062
Creatinine, mg/dl	0,8 ± 0,1	0.8 ± 0.1	0.8 ± 0.1	0.338
Hemoglobin, mg/dl	14.7 ± 1.8	14.4 ± 2.1	14.2 ± 1.7	0.103
Endocane, pg/ml	559 ± 234	555 ± 223	331± 252	<0.001
CRP, mg/dl	3.1 (0–10)	3.2 (0–10)	1.7 (0–9)	0.256
SBP mmHg	129 ± 17	124 ± 16	125 ± 13	0.299
DBP, mmHg	78 ± 11	75 ± 9	77 ± 9	0.145
LAD TIMI-FC	32.5 ± 6.8	19.8 ± 0.9	19.8 ± 0.8	<0.001
CX TIMI-FC	22.3 ± 8.6	18.1 ± 0.8	17.8 ± 0.8	<0.001
RCA TIMI-FC	27.2 ± 9.9	17.4 ± 0.75	17 ± 0.8	<0.001
Mean TIMI- TF	27.3 ± 4.2	18.4 ± 0.6	18.2 ± 0.6	<0.001

CSF: coronary slow flow; CAD: coronary artery disease; ACEI/ARB: angiotensin-converting enzyme inhibitors/angiotensin receptor blockers; ASA: acetyl salicylic acid; BMI, body mass index; CCB, calcium channel blocker; CRP: C-reactive protein; DBP: diastolic blood pressure; HDL: high-density lipoprotein; GFR: glomerular filtration rate; LAD: left anterior descending; LDL: low-density lipoprotein; RCA: right coronary artery; SBP: systolic blood pressure; TIMI-FC: TIMI frame count.

Serum endocan levels were positively but weakly correlated with total cholesterol (r=0.193; p=0.012) and LDL-cholesterol (r=0.167; 0.035). Endocan and mean TIMI-FC were positively correlated (r=0.267; p<0.001). Mean TIMI-FC was positively and weakly correlated with uric acid and creatinine. There was no difference in endocan levels between the CSF and CAD groups; therefore, we considered the presence of the CSF or CAD group as a single dependent variable and then performed a logistic regression analysis. The presence of hyperlipidemia (OR: 2.701 [2.011–5.678]; p=0.039) and endocan levels (OR=1.984 [1.319–2.578]; p<0.001) was found to be significant variables for the presence of CSF and CAD. In the logistic regression analysis between the CAD and control groups, the variables predicting the presence of CAD were again hyperlipidemia (OR=4.643 [2.861–7.877]; p<0.001) and endocan levels (OR=2.235 [1.434–2.784]; p<0.001) (Table 2). On ROC analysis, endocan levels above 450 pg/mL predicted the presence of CAD and/or CSF with 75% sensitivity and 60% specificity (AUC=0.779 [0.690–0.850], p<0.001).

**Table 2:** Univariate and multivariate logistic regression analysis.

Dependent variable: Presence of CSF				
		Beta ± standard deviation	Odds ratio and 95% confidence Interval	p-value
**Univariate**				
	Age	0.23±0.22	0.978 (0.916–1.356)	0.348
	Diastolic blood pressure	0.25±0.23	1.674 (0.946–1.564)	0.349
	Glucose	0.001±0.07	0.912 (0.767–1.347)	0.102
	Hyperlipidemia	0.11±0.08	3.564 (2.061–6.877)	0.025
	Beta blocker	0.45±0.68	1.578 (0.608–3.207)	0.749
	ASA	0.36±0.67	3.789 (1.298–6.567)	0.018
	Statin	0.77±0.53	6.712 (2.386–11.342)	<0.001
	GFR	0.04±0.02	1.457 (0.798–1.347)	0.206
	Uric asid	0.143±0.64	1.236 (0.856–1.956)	0.459
	Endocan	0.001±0.001	1.890 (1.546–2.678)	<0.001
**Multivariate**				
	Hyperlipidemia	0.11±0.08	4.643 (2.861–7.877)	0.025
	Endocan	0.001±0.002	1.235 (1.434–2.784)	<0.001

2 GFR: glomerular filtration rate.

## DISCUSSION

The most important findings of our study were as follows: (1) 2.6% of the population we screened during our study had CSF; (2) both the CSF and CAD groups had higher endocan levels than the control group; (3) there were similar endocan levels between the CSF and CAD groups; (4) endocan levels were positively correlated with the mean TIMI-FC; and (5) serum endocan levels (especially values ≥450 pg/mL) and the presence of hyperlipidemia were the main predictors of the presence of both CSF and CAD.

In a previous meta-analysis of 15 studies^5^, high endocan levels were also associated with cardiovascular diseases. The results of our study were consistent with the meta-analysis published in the literature. As the age, gender, diabetes, smoking, and hypertension rates of the control group and the other study groups were similar in the study population, only hyperlipidemia was found to be a predictive clinical feature for the presence of CSF and/or CAD. However, other cardiovascular risk factors other than hyperlipidemia were not found to be the main predictors of CSF and CAD.

In our study, CRP and endocan were not correlated. Although they are inflammatory markers, we think that the lack of correlation between endocan and CRP is related to their use of different inflammatory pathways^12-16^. In our study, CRP values were similar in all three groups. The reason why CRP values were not different between the CSF and CAD groups is as follows. First, we excluded acute or chronic infection in our study. Second, other cardiovascular risk factors, except for hyperlipidemia in the control group, were similar in the CSF and CAD groups. In this study, endocan levels predicted cardiovascular disease much better than CRP.

A few small population case/control studies show that endocan levels are correlated with increased blood pressure in hypertensive patients^17,18^. When confounding factors such as the prevalence of micro atheroma accompanying hypertension are taken into account, our findings seem potentially accurate.

Studies show that uric acid is elevated in patients with CSF. Increased uric acid levels show proinflammatory properties and vascular endothelial damage occurs more rapidly at levels higher than normal in uric acid levels^19^. In our findings, the mean TIMI-FC and uric acid values were weakly correlated and were compatible with the literature. In addition to uric acid, we found a correlation between creatinine values and mean TIMI-FC.

Our study has some limitations. In the control group, cardiovascular risk factors were similar except for hyperlipidemia. This strengthened our study. The reason for this may be that we are a tertiary center and the patients included in coronary angiography are selected from those who are symptomatic or present with some findings indicating ischemia, as well as those with additional cardiovascular risk factors. Therefore, the association between endocan and other cardiovascular risk factors may have been statistically blunted. If we had also analyzed the levels of some cytokines such as IL-1 or IL-6, which are involved in the NF-κB pathway, we could have better evaluated the correlations of endocan with inflammatory cytokines involved in atherosclerosis.

## CONCLUSION

Increased endocan levels (≥450 pg/mL), especially in asymptomatic patients with cardiovascular risk factors, may be a good biomarker for further investigations. Additional studies are needed to clarify its value. Endocan levels are not only elevated in CSF and CAD but also associated with CSF and CAD. Therefore, it appears to be a potent proinflammatory proteoglycan in atherosclerosis.
